# The Roles of PINK1 and Parkin in Parkinson's Disease

**DOI:** 10.1371/journal.pbio.1000299

**Published:** 2010-01-26

**Authors:** Rachel Jones

**Affiliations:** Freelance Science Writer and Editor, Welwyn, Hertfordshire, United Kingdom

**Figure pbio-1000299-g001:**
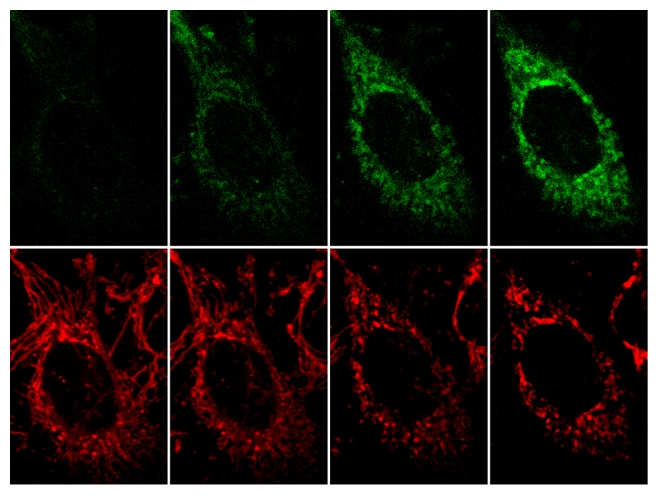
PINK1 (labeled in green by GFP, top panels) accumulates on mitochondria (labeled in red with mitotracker, bottom panels) when they are uncoupling with CCCP for 60 minutes or otherwise impaired, signalling mitochondrial dysfunction to Parkin, which triggers their elimination.

[Fig pbio-1000299-g001]Research into the causes of both sporadic and familial Parkinson's disease have led to the idea that a key risk factor might be mitochondrial dysfunction. The neurons of the substantia nigra, which are specifically lost in Parkinson's disease, seem to be especially vulnerable to the effects of mitochondrial damage. Recent work showed that the protein Parkin, which is mutated in some forms of familial Parkinson's disease, is recruited from the cytoplasm to damaged mitochondria and that this leads to the breakdown of the mitochondria by processes acting within the cell (autophagy).

The mitochondrial membrane protein PINK1 is also mutated in some forms of familial Parkinson's disease. In a new study in this issue of *PLoS Biology*, Youle and colleagues address the question of how Parkin recognises damaged mitochondria, and they find a crucial role for PINK1.

Damaged mitochondria cannot maintain the potential difference across their inner membrane. This membrane depolarization can be mimicked experimentally by treating cells with CCCP—a “protonophore” that pokes a hole in the membrane, making it more permeable to protons. To investigate the role of PINK1 in the cell's response to mitochondrial damage, the authors treated cultured cells with CCCP and found that PINK1 accumulated on the depolarized mitochondria over several hours. In cells that contained some normal, polarized mitochondria and some depolarized ones, PINK1 accumulated specifically on the depolarized mitochondria and Parkin was recruited to those same damaged mitochondria.

PINK1 might accumulate in damaged mitochondria because of increased synthesis and/or reduced degradation of the protein. To investigate which mechanism might be responsible, the authors first allowed PINK1 to accumulate in the presence of CCCP for three hours. They then washed out the CCCP and saw a rapid reduction in the amount of PINK1, suggesting that PINK1 degradation was inhibited when the mitochondria were depolarized. Addition of a proteasome inhibitor to untreated cells caused the accumulation of a cleavage product of PINK1 that was also found at high levels after CCCP washout in the inhibitor-treated cells, but at lower levels during CCCP treatment. This is consistent with the accumulation of PINK1 on depolarized mitochondria being due, at least partly, to reduced degradation of PINK1. By contrast, the authors found that depolarization of mitochondria by CCCP did not lead to increased transcription of PINK1. They propose a two-step model for PINK1 processing in which the first PINK1 cleavage step is voltage-dependent but proteasome-independent and is followed by complete degradation of the cleavage product by the proteasome.

Youle and colleagues found that Parkin was not required for accumulation of PINK1 on depolarized mitochondria, but that Parkin could not be recruited to depolarized mitochondria in the absence of PINK1. In addition, whereas expression of ectopic Parkin could induce autophagic destruction of depolarized mitochondria in normal cells, it could not do so in cells lacking PINK1. These findings indicate that PINK1 accumulates on depolarized mitochondria before Parkin and that both are required for the subsequent autophagy of mitochondria.

The authors induced ectopic overexpression of PINK1 and found that this accelerated the recruitment of Parkin and loss of mitochondria in the presence of CCCP. They then generated a chimeric protein that had the active part of PINK1 and a membrane anchor from another protein, but that lacked a cleavage site. As expected, this protein was more stable than wild-type PINK1. Its presence led to the recruitment of Parkin to mitochondria that were not depolarized and also to increased mitochondrial autophagy. Excessive PINK1 accumulation on mitochondrial membranes is therefore sufficient to recruit Parkin and activate mitochondrial autophagy, even in the absence of membrane depolarization.

A variety of mutations in the *PINK1* and *Parkin* genes cause early-onset Parkinson's disease in humans. This paper reports that these mutations disrupt distinct steps in Parkin recruitment to mitochondria and induction of autophagy. It is easy to imagine that this could lead to accumulation of damaged mitochondria in cells. The increased vulnerability of the neurons in the substantia nigra to mitochondrial damage might be because greater oxidative stress is found here than elsewhere in the brain and/or because neurons do not regenerate by division and turnover as do other cell types.

These findings provide important insights into how Parkin and PINK1 function in normal cells and clues to how mutations might lead to Parkinson's disease. Further investigations of mitochondrial turnover in the neurons of the substantia nigra should lead to a fuller understanding of this process and might identify potential therapeutic targets for the treatment of the disease.

**Narendra DP, Jin SM, Tanaka A, Suen D-F, Gautier CA, et al. (2010) PINK1 Is Selectively Stabilized on Impaired Mitochondria to Activate Parkin. doi:10.1371/journal.pbio.1000298**

